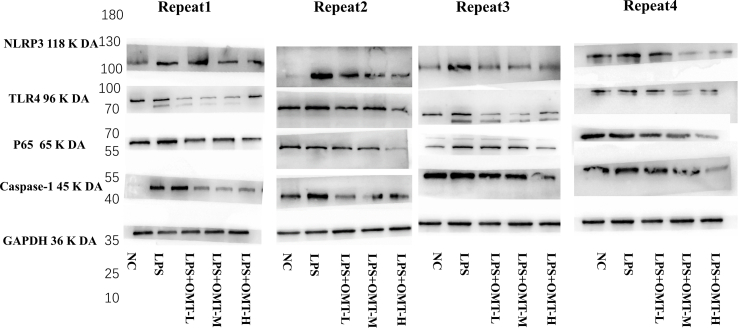# Oxymatrine blocks the NLRP3 inflammasome pathway, partly downregulating the inflammatory responses of M1 macrophages differentiated from THP-1 monocytes^☆^

**DOI:** 10.1016/j.bbrep.2023.101482

**Published:** 2023-05-09

**Authors:** Ke Zhang, Youyang Liu, Yunlu Zhao, Qi Guo, Shengjun An, Shuhui Wu

**Affiliations:** a School of Basic Medicine, Hebei University of Chinese Medicine, Shijiazhuang, 050200, Hebei, China; b Department of Cardiovascular Diseases, Shinnshu University Shinshu University School of Medicine, Matsumoto, 390-8621, Japan; c Department of Molecular and Cellular Physiology, Shinshu University School of Medicine, Matsumoto, 390-8621, Japan; d Hebei Provincial Engineering Laboratory of Plant Bioreactor Preparation Technology, No. 326 Xinshi South Road, Qiaoxi District, Shi Jiazhuang, 050090, Hebei, China

**Keywords:** Oxymatrine, NLRP3 inflammasome, Macrophages, Inflammation

## Abstract

Many chronic inflammatory diseases, such as autoimmune inflammation, are associated with M1 macrophages, and the key to their treatment is blocking inflammation. Oxymatrine (OMT), a traditional Chinese medicine, has a marked anti-inflammatory effect. However, its anti-inflammatory target and mechanism in M1 cells remain unclear, which limits its clinical application. In this study, we investigated the anti-inflammatory effects of oxymatrine (OMT) on the M1 inflammatory response. We also determined the relationship between OMT treatment and the nucleotide-binding oligomerization domain-like receptor protein 3 (NLRP3) pathway with OMT treatment. To this end, we induced the differentiation of human peripheral blood monocytes (THP-1) into M1 cells. THP-1 cells were induced with a phorbol ester (phorbol-12-myristate-13-acetate (PMA)) and differentiated into naïve M0 macrophages. M0 cells were induced into M1 cells using lipopolysaccharide (LPS). The experimental groups were divided into the M0 macrophage group (NC), M1 inflammatory response group (LPS group), and M1 group treated with different concentrations of OMT (LPS + OMT-L, LPS + OMT-M, LPS + OMT-H). The cells in the OMT-treated groups were treated with OMT for 6 h, followed by LPS for 24 h, and the LPS group was treated with LPS only. The resulting supernatants and cells were collected. The secretion levels of NO were detected by the Griess method and the secretion levels of TNF-α and IL-1β in the supernatants were detected by the ELISA method. The secretion levels of these inflammatory factors were reduced in every OMT-treated group compared to the LPS group (P < 0.01), and the most significant reductions were found in the OMT-H group (P < 0.0001). By western blotting, the protein expression levels of TLR4, NF-κB, NLRP3, and Caspase-1 were all found to be downregulated in the cells of OMT-treated groups compared to the LPS group (P < 0.0001). *In situ* changes in NLRP3 expression were observed using immunofluorescence. The fluorescence intensity of NLRP3 in M1 cells was weaker in all OMT intervention groups than in the LPS group (P < 0.001). In conclusion, OMT has significant anti-inflammatory effects on the M1 inflammatory responses, and the TLR4/NF-κB/NLRP3 pathway was blocked proportional to the concentration of OMT.

## Highlights

• The present study was designed to investigate the impact of Oxymatrine on M1 macrophages.
• The Oxymatrine was effective in downregulating the inflammatory reaction of M1 through the NLRP3 inflammasome pathway.
• Oxymatrine significantly reduced pro-inflammatory factor secretion and downregulated the NLRP3 pathway in M1 macrophages.

## 1 Introduction

Macrophages play an important role in chronic inflammatory diseases, such as atherosclerosis and autoimmune diseases [1,2]. Macrophages are highly heterogeneous, with naïve M0 macrophages differentiating into M1 or M2 cells in different microenvironments [3]. Grosjean et al. and Gordon et al. have both reported that M1 macrophages have a pro-inflammatory effect, whereas M2 macrophages have an anti-inflammatory effect [4,5]. Therefore, blocking the inflammatory pathway in M1 macrophages is a potential treatment strategy for alleviating inflammation. NOD-like receptors (NLRs) are important pattern-recognition receptors (PRRS) in innate immune cells. The nucleotide-binding oligomerization domain-like receptor protein 3 (NLRP3) inflammasome has been confirmed to play a key role in chronic inflammation, and its formation triggers the activation of the Caspase-1/IL-1β signaling cascade, which can amplify the inflammatory response [6].

Several studies conducted by Piao et al. and Chen et al. reported on several Chinese herbal medicines, such as triptolide [7] and sinomenine [8,9]. The traditional Chinese medicine monomer oxymatrine (OMT) has a tetracyclic quinoline structure, which has a variety of pharmacological effects, such as anti-inflammatory, free radical scavenging, antitumor activity, and improved insulin resistance. However, its therapeutic mechanism remains unknown [10], and its effect on the NLRP3 inflammasome is poorly understood [11]. Therefore, in this study, we investigated the anti-inflammatory effect of OMT on TLR4/NF-ΚB and NLRP3 inflammasomes in M1 cells.

## 2 Materials and methods

### 2.1 Cell culture

Human peripheral blood monocytes (THP-1) were donated by the Immune Laboratory of Hebei Medical University. The cells were incubated in RPMI-1640 medium (Gibco, USA) containing 10% fetal bovine serum (FBS) (Gibco, USA) and 1% penicillin streptomycin (Sigma, USA) at 37 °C in a 5% CO_2_ incubator. After counting using the blood cell counting board, the cells were subcultured at a ratio of 1:3 and selected during the logarithmic growth period for subsequent experiments.

### 2.2 Dose- and time-effect analysis of PMA (phorbol-12-myristate-13-acetate)

Using a Cell Counting Kit-8 (CCK8) (Share Bio, Shanghai, China) assay, cell viability curves were established for the dose- and time-dependent effects of phorbol 12-myristate 13-acetate (PMA) (Multi Sciences, Hangzhou, China) on THP-1 cells.

THP-1 cells were inoculated in a 96-well plate, at a density of 1 × 10^4^ cells/well and treated with different concentrations of PMA (0, 20, 50, 100, and 200 ng/ml)/well for 24 h to determine the dose effect. According to CCK-8 kit instructions, the absorbance of each well was measured according to the manufacturer's instructions. Cell viability was calculated using the following formula: cell viability (%) = (Abs 450 nm of administered cells – Abs blank group)/(Abs negative group – Abs blank group) × 100.

To determine the time effect of PMA, THP-1 cells were seeded in 96-well plates and the optimal PMA concentrations were determined by the dose-effect curve. Cell viability was measured at different time points (0, 12, 24, and 48 h) using the CCK-8 assay, and cell viability was calculated to obtain a time-effect curve.

### 2.3 M0 cell identification

THP-1 cells in the subculture in the logarithmic growth period were inoculated in 12-well glass bottom plates at 5 × 10^5^ cells/well and induced under the optimal concentration and action time of PMA. M0 cells were identified by observing the cell morphology and immunofluorescence staining. The cell morphology was observed using a light microscope (E-CLIPSE Ts2; Nikon, Japan). For immunofluorescence staining, the cells were fixed with 4% paraformaldehyde (PFA) (Biosharp, Anhui, China) and incubated with diluted rabbit primary antibody against CD68 (1:300) (Boster, Wuhan, China) overnight at 4 °C. The sections were incubated with a secondary antibody (rabbit, 1:300; Absin, Shanghai, China) in the dark at room temperature for 1 h on the following day. Antifade Mounting Medium with DAPI (Servicebio, Wuhan, China). Slides were analyzed under a fluorescence microscope (Zeiss Imager Z2; Zeiss, Germany).

### 2.4 Establishment and identification of M1 cell model

After THP-1 differentiation into M0 cells at the optimal PMA concentration and action time, PMA was removed. Then lipopolysaccharide (LPS) (1 μg/ml) (Solarbio, Beijing, China) was added for 24 h, and adenosine triphosphate (ATP) (5 mM) (Aladdin, Shanghai, China) was added for 30 min. M1 cell morphology was observed under a light microscope (E-CLIPSE Ts2; Nikon, Japan). A rabbit primary antibody against CD86 (1:300) (Boster, Wuhan, China) was used to identify M1 cells using the protocol described in section 2.3.

### 2.5 Dose- and time-effect analysis of OMT

After the differentiation of THP-1 cells into M0 cells with the optimal PMA concentration and action time cultured with 96-well plate described in section 2.2, PMA was removed and different concentrations of oxymatrine (OMT) (Nanjing Dilger Medical Technology, Nanjing, China) (0.25, 0.5, 1, 2, and 8 mg/ml) were added to the cells for 6 h. For the time-effect analysis, the cells were treated with 0.5, 1, and 2 mg/ml OMT, and a CCK8 assay was established at different time points (0, 6, 12, 24, and 48 h). The calculation formula of cell viability is the same as described in section 2.2.

### 2.6 Experimental groups of OMT treatment

The experimental groups of OMT treatment were as follows: M0 macrophage group (NC), M1 macrophage group (LPS group), and OMT-treated groups (LPS + OMT-L, LPS + OMT-M, and LPS + OMT-H at concentrations of 0.5, 1, and 2 mg/ml).

After M0 differentiated from THP-1 by PMA (as described in section 2.2), the cells of OMT-treated groups were added with different concentration of OMT for 6 h, followed by LPS 1 μg/ml for 24 h and ATP 5 mM for 30 min. Then, the LPS group was added to the LPS-treated group. The culture conditions used were selected as described in sections 2.7 to 2.10.

### 2.7 Griess method

THP-1 cells were seeded in 6-well plates at a density of 5 × 10^5^ cells/well. After the cells are treated with PMA, OMT, and LPS (as described in section 2, 2.2.6), nitrous oxide (NO) was detected from the supernatant of each well using a NO detection kit (Beyotime Biotechnology, Shanghai, China), according to the manufacturer's instructions. Briefly, 50 μl of supernatant of each well was used for detection, for which standard samples of NaNO_2_ (50 μl; 0, 1, 2, 5, 10, 20, 40, 60, and 100 μM) were added into 96-well plates. Next, Griess reagents I and II were added, and the cells were incubated at room temperature for 10 min. The absorbance at 540 nm was assayed with a multimode microplate reader (Varioskan LUX; Thermo Fisher Scientific, USA) [12].

### 2.8 Enzyme-linked immunosorbent assay

The THP-1 cells were inoculated into 12-well glass bottom plates with 5 × 10^5^ cells/well, and the cells were treated under the conditions described in section 2, 2.2.6. The TNF-α and IL-1β concentrations in the supernatant were assayed using an ELISA detection kit (Multi Sciences, China) according to the manufacturer's instructions. Absorbance was measured at 450 and 570 nm using a multimode microplate reader (Varioskan LUX; Thermo Fisher Scientific, USA).

### 2.9 Western blot analysis

The THP-1 cells were inoculated into 6-well plates with 5 × 10^5^ cells/well, and cell treatment was conducted as described in section 2.2 and 2.6. The collected protein samples of each group were added RIPA Lysis buffer (Share bio, Shanghai, China) for 30 min. The protein concentration was measured using bicinchoninic acid (Thermo Fisher Scientific, USA). Briefly, 20 μg of protein sample were separated by 7.5% SDS-PAGE (Epizyme, Shanghai, China) at 80 V for 30 min and 120 V for 50 min before transferring to a PVDF membrane (Merck, Germany) at 300 mA for 1 h. The membranes were blocked with 5% nonfat milk in TBST (Tris-buffered saline containing 0.05% Tween-20) for 2 h at room temperature (RT). The membranes were then incubated with rabbit primary antibodies against TLR4 (1:1000) (Servicebio, Wuhan, China), NF-κB (1:1000) (Servicebio, Wuhan, China), NLRP3 (1:1000) (Abcam, Jiangsu, China), Caspase-1 (1:1000) (Abways Biotechnology, Shanghai, China), and GAPDH (1:1000) (Abbkine, Wuhan, China) overnight at 4 °C. The next day, the membranes were washed three times for 5 min with TBST and incubated with goat anti-rabbit secondary antibodies (1:5000) (Mei5bio, Beijing, China) for 1 h at RT. Visualization was performed using a chemiluminescence instrument (Fusion FX5 Spectra, France) with an ECL solution (Biosharp, China). GAPDH was used as an internal reference, and the gray value was obtained using ImageJ (version 1.8.0).

### 2.10 Immunofluorescence staining of NLRP3

THP-1 cells were inoculated into 12-well glass bottom plates at a density of 5 × 10^5^ cells/well. Cell treatment was conducted as described in section 2.2, 2.6. Rabbit primary antibody against NLRP3 (1:300) (Affinity, Jiangsu, China) was used. The staining protocol was the same as that described in section 2.3. Lastly, the slides were analyzed under a fluorescence microscope (Zeiss Imager Z2), and the average fluorescence intensity was obtained using ImageJ (version 1.8.0).

### 2.11 Statistical analysis

GraphPad Prism (version 9.0) was used for statistical analysis. All data were expressed as the mean ± standard error of the mean (SEM) (n ≥ 3) unless otherwise indicated. One-way analysis of variance (ANOVA) was conducted, followed by Tukey's multiple comparison tests for the comparison of multiple groups. Statistical significance is indicated as *P < 0.05, **P < 0.01, ***P < 0.001, or ****P < 0.0001.

## 3 Results

### 3.1 Time- and dose-effects of PMA and OMT

As previously reported by Zhou et al., the cell adhesive effect depended on the PMA concentration, with the most substantial effect observed at 40 nM (i.e., 24 ng/ml) [13]. In our study, the CCK8 results showed that compared with the control group, cell viability decreased in relation to PMA concentration. Considering its cytotoxicity, 20 ng/ml PMA was selected for further experiments.

**Fig. 1:**
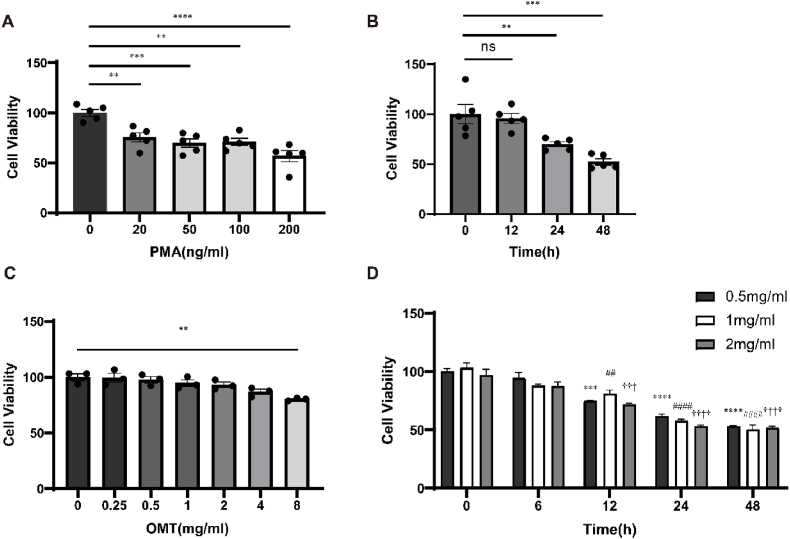
Time- and dose-effect analysis of PMA and OMT. (A) The viability of the PMA-induced THP-1 cells at different concentrations (0, 20, 50, 100, and 200 ng/ml) was determined by CCK8 assay. (B) The viability of the PMA-induced THP-1 cells at a concentration of 20 ng/ml at different time points (0, 12, 24, and 48 h) was determined by CCK8 assay. (C) The viability of M0 macrophages at different concentrations (0, 0.25, 0.5, 1, 2, 4, and 8 mg/ml) of OMT was determined by CCK8 assay. (D) The viability of M0 macrophages at 0.5, 1, and 2 mg/ml OMT at different time points (0, 6, 12, 24, and 48 h) was determined by CCK8 assay. All data are shown as the mean ± SEM (n = 3). **P < 0.01, ***P < 0.001, ****P < 0.0001, ^##^P < 0.01, ^####^P < 0.0001, ^†††^P < 0.001, ^††††^P < 0.0001. vs 0 mg/ml or 0 h group.

Moreover, after 24 h, 20 ng/ml PMA affected THP-1 cells, which were entirely adherent, with a viability of approximately 70%. However, the cell viability decreased to 60% after 48 h of treatment (Fig. 1B). Therefore, the PMA-acting time was determined as 24 h for the induction of THP-1 cells into M0.

After 6 h of the treatment of on M0 cells with different concentrations of OMT, the cell viability did not change compared to that of the control group, except at 8 mg/ml (Fig. 1C). With regards to the time-effect analysis, the cell viability was significantly decreased after 12, 24, and 48 h of 0.5, 1, and 2 mg/ml OMT treatment, except for the 6 h treatment. Therefore, 0.5, 1, and 2 mg/ml OMT were chosen as the low (L), medium (M), and high (H) concentrations, respectively. Additionally, a treatment time of 6 h was selected for further experiments.

### 3.2 Morphological observation and identification of M0 and M1 macrophages

**Fig. 2:**
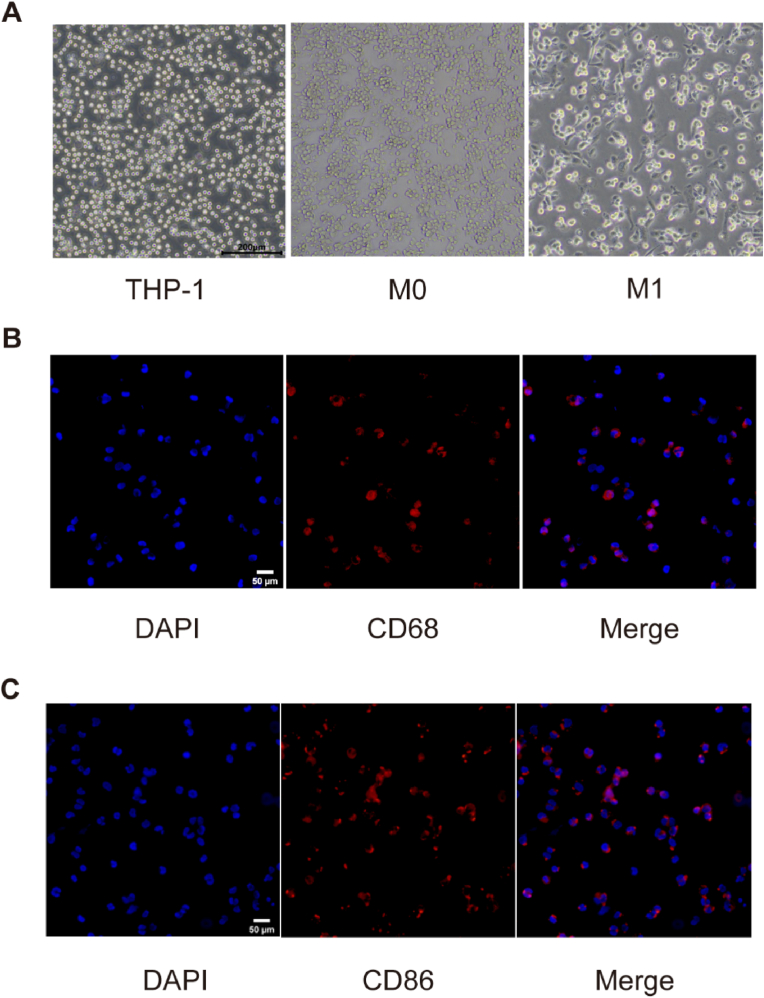
Morphological images of cells in different states and identification of M0 and M1 macrophages. (A) Morphology of THP-1 cells, M0 macrophages, and M1 macrophages. Scale bar: 200 μm. THP-1 monocytes were round, translucent, and suspended; M0 macrophages were clustered and adherent; M1 macrophages were adherent and can extend pseudopodias. (B) Immunofluorescence staining on M0 cells. Scale bar: 50 μm. Colocalization of CD68 (red) and DAPI (blue). (C) Immunofluorescence staining of M1 cells. Scale bar: 50 μm. Colocalization of CD86 (red) and DAPI (blue). (For interpretation of the references to colour in this figure legend, the reader is referred to the Web version of this article.)

The statuses of THP-1 cells and M0 and M1 macrophages were observed using an inverted phase-contrast microscope. THP-1 monocytes, M0, and M1 are shown in Fig. 2A. The M0 and M1 cells changed from suspended to adhered with various and irregular shapes. M0 and M1 cells were detected using a CD68/CD86 fluorescent antibody and all cells were positive (Fig. 2B and C).

### 3.3 OMT treatment reduced the expression levels of NO, TNF-α, and IL-1β in the M1 culture supernatant

**Fig. 3:**
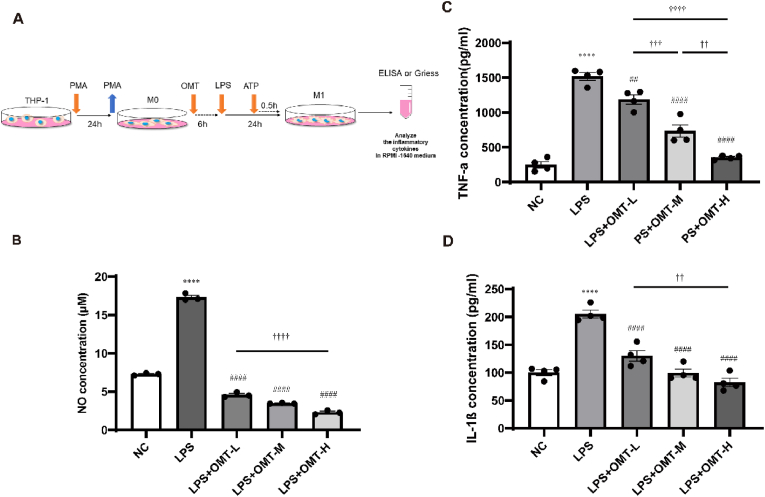
Effects of OMT (L, M, H) on NO, TNF-α, and IL-1β levels in the cell supernatant of M1 macrophages in each treatment group. (A) The experimental procedures for the OMT intervention M1 inflammatory response. (B) The NO concentration in cell supernatant of each group were assayed by Griess. (C–D) The effects of OMT on the TNF-α and IL-1β levels in cell supernatant of each group were assayed by enzyme-linked immunosorbent assay. All data are shown as the mean ± SEM (n = 3). ****P < 0.0001 vs NC group; ^##^P < 0.01, ^####^P < 0.0001 vs LPS group; ^††^P < 0.01, ^†††^P < 0.001, ^††††^P < 0.0001: pairwise comparison among OMT (L, M, H) intervention groups.

THP-1 cells were used to evaluate the anti-inflammatory effects of OMT on the M1 inflammatory response (Fig. 3A). Using the Griess method, the NO levels in the LPS group were significantly higher than those in the NC group (Fig. 3B), and similar changes in the TNF-α and IL-1β levels were detected by ELISA (Fig. 3C). Compared with the LPS group, the levels of NO, TNF-α, and IL-1β in the LPS + OMT-L, LPS + OMT-M, and LPS + OMT-H groups all decreased, which suggests that OMT had a significant blocking effect on M1 inflammatory response.

### 3.4 OMT downregulated the expression of proteins associated with the inflammatory pathway in M1 macrophages

**Fig. 4:**
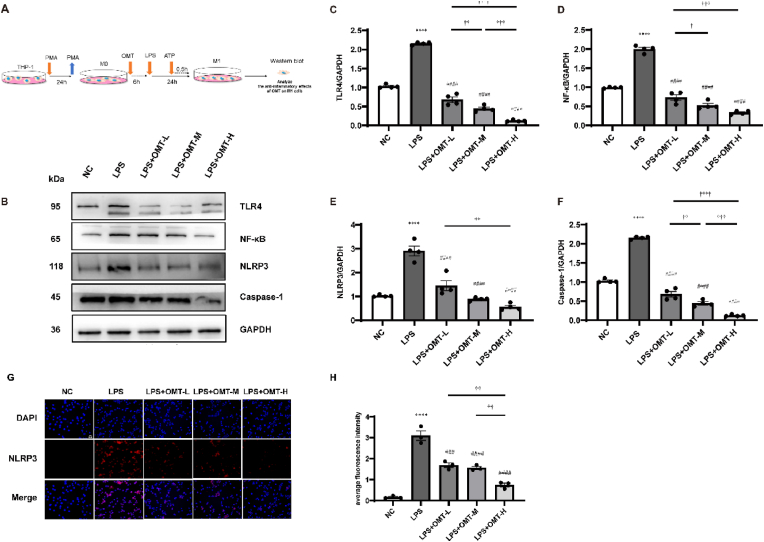
Protein expression of TLR4, NF-κB, NLRP3, and Caspase-1 in M1 macrophages detected by western blotting. (A) The experimental procedures for inflammatory pathway proteins of M1 macrophages. (B) Representative figures of the protein expression of TLR4, NF-κB, NLRP3, and Caspase-1 in M1 macrophages. (C–F) Densitometry analyses were used to quantify TLR4, NF-κB, NLRP3, and Caspase-1 in M1 macrophages (n = 4). (G–H) NLRP3 immunofluorescence staining of each group. Red indicates NLRP3; blue indicates DAPI-stained nucleus. Scale bar: 50 μm (n = 3). All data are shown as the mean ± SEM. ****P < 0.0001 vs NC group; ^####^P < 0.0001 vs LPS group; ^†^P < 0.05, ^††^P < 0.01, ^†††^P < 0.001, ^††††^P < 0.0001: pairwise comparison among OMT (L, M, H) intervention groups. (For interpretation of the references to colour in this figure legend, the reader is referred to the Web version of this article.)

The protein expression levels of TLR4, NF-κB, NLRP3, and Caspase-1 were detected by western blotting (Fig. 4B–F), which are representative of repeated experiments (Fig. 4B). Compared to the LPS group, the protein expression levels of the four indices of M1 in the LPS + OMT-L, LPS + OMT-M, and LPS + OMT-H groups were significantly decreased. Meanwhile, significant decreases were observed in the LPS + OMT-H group compared to the LPS + OMT-L and LPS + OMT-M groups, indicating that OMT could block the inflammatory pathway of M1 in a concentration-dependent manner.

### 3.5 Immunofluorescence of NLRP3

Compared to the LPS group, the expression of NLRP3 was significantly reduced in the LPS + OMT-L, LPS + OMT-M, and LPS + OMT-H groups, further demonstrating the blocking effect of OMT on the M1 inflammatory response.

## 4 Discussion

As a cell population with plasticity and pluripotency, macrophages can be divided into the M1 and M2 types, which have different functions [14]. M2 macrophages have an anti-inflammatory effect, while M1 presents as a pro-inflammatory phenotype whose function is mainly activated by LPS and IFN-γ, secreting various inflammatory mediators, including tumor necrosis factor (TNF-α), IL-1β, and IL-6. In addition, the M1 type has the function of presenting antigen and firing immune response and promoting inflammation. In a previous study, Linden et al. found that exercise inhibited the infiltration of M1 macrophages and promoted the polarization and recruitment of M2 macrophages, thereby improving obesity-induced insulin resistance [15]. Similarly, Yang et al. showed that regulating macrophage polarization and changing the proportion of macrophage phenotypes in plaques could affect the development of atherosclerosis [16]. Therefore, the modulation of macrophage polarization is a strategy for the treatment of inflammatory diseases.

M1 macrophages are involved in the development of local inflammatory responses in chronic inflammatory diseases, such as type 2 diabetes mellitus [17], atherosclerosis [18], and Alzheimer's disease [19]. Therefore, exploring how to block the inflammatory response of macrophages for therapeutic intervention against macrophage-mediated pathologies is necessary and meaningful work.

The inflammatory response of M1 macrophages is mainly mediated by the activation of the inflammasome and the induction of cells to secrete proinflammatory factors, wherein Nod-like receptor protein 3 (NLRP3) is of great concern in chronic inflammation [20]. Inflammation driven by the abnormal activation of NLRP3 is the basis of many chronic degenerative diseases; thus, the inhibition of NLRP3 is a potential strategy for the treatment of inflammatory diseases [21]. Schunk et al. revealed that NLRP3 inflammasome activation increases systemic inflammation, thus promoting the development of atherosclerosis [22].

The NLRP3 inflammasome is a cytosolic protein containing three structural domains [23]. These include NLRP3, an adapter protein called PYCARD or ASC, and zymogen pro-Caspase-1. The third domain is a pro-inflammatory messenger protein that promotes the release of IL-1β and IL-18 [24]. NLRP3 and IL-1β transcription are first initiated by TLRs and the induction of cytokines, after which the NLRP3 inflammasome aggregates via PAMPs and DAMPs, leading to the activation of Caspase-1, which promotes the maturation and release of the inflammatory cytokines, including Caspase-1 and IL-1β. NLRP3 can be activated by various abnormal metabolites, such as bacterial toxins, extracellular ATP, and cholesterol [25], whereas in human monocytes, the initiation element alone is sufficient to mediate both Caspase-1 activation and IL-1β release [26]. Therefore, in this study, THP-1 cells were inducted by PMA to transform monocytes into M0 macrophages, which were later induced into M1 macrophages using lipopolysaccharide (LPS) and adenosine triphosphate (ATP) to establish an inflammation model. This model was then used to identify drugs that block the NLRP3 inflammasome for therapeutic intervention against chronic inflammatory diseases.

Herein, we were also interested in investigating anti-inflammatory mechanisms, and found that oxymatrine (OMT) had a tetracyclic quinoline structure, which can play an anti-inflammatory role. At present, research on the action target of OMT is scarce [10]. To determine whether this drug can be widely used in clinical practice, research on its efficacy and anti-inflammatory targets is necessary. Therefore, in the study, we analyzed the protein expression changes in the inflammatory pathway in M1 blocked by OMT, including TLR4/NF-κB/Caspase-1 and NLRP3, as well as cell-secreted components, such as NO, TNF-α, and IL-1β.

By focusing on the NLRP3 inflammasome pathway, we found that NO, TNF-α, and IL-1β in the LPS group were significantly higher than those in the NC group, indicating that LPS activated the inflammatory response of macrophages. Compared with the LPS group, the levels of NO, TNF-α, and IL-1β in the OMT intervention groups all decreased, indicating that OMT significantly inhibited the inflammatory response of macrophages induced by LPS. These findings indicate that OMT is an effective anti-inflammatory drug.

Our results also showed that the TLR4/NF-κB signaling pathway is involved in the regulation of inflammatory response, and TLR4/NF-κB can further activate the NLRP3 inflammasome to induce the release of pro-inflammatory factors, thereby aggravating chronic inflammation, In this study, compared with the LPS group, the expression levels of TLR4, NF-κB, NLRP3, and Caspase-1 proteins in the OMT intervention group were decreased, indicating that OMT can reduce the inflammatory response of macrophages by inhibiting NLRP3, indicating that NLRP3 is a target site of OMT. At the same time, the levels of inflammatory pathway proteins in the high concentration intervention group were found to be lower than those in the medium- and low-concentration intervention groups, indicating that the anti-inflammatory effects of OMT are dose-dependent.

In conclusion, we found that OMT can significantly block the inflammatory response of M1 cells by blocking the TLR4/NF-κB/NLRP3 pathway to a certain extent in a dose-dependent manner. This study not only elucidates the mechanism by which OMT blocks the M1 inflammatory reaction, but also establishes a new anti-inflammatory target of OMT, namely NLRP3, which provides an experimental basis for the clinical treatment of chronic inflammatory diseases involving M1 cells.

## Limitation of the study

A limitation of this study is the lack of investigation into the anti-inflammatory effects of OMT on specific types of chronic diseases. The experimental concentration of PMA in this study was 20 ng/ml, but there may be a more efficient and less cytotoxic concentration below 20 ng/ml.

## Author contributions

**Ke Zhang:** conceptualization, investigation, writing ‒ original draft; **Youyang Liu:** investigation, validation; **Yunlu Zhao:** formal analysis, writing ‒ review and editing; **Qi Guo:** visualization, writing ‒ Review and Editing; **Shengjun An:** methodology, resources; **Shuhui Wu:** methodology, resources, writing ‒ review and editing.

## Declaration of competing interest

The authors declare that they have no known competing financial interests or personal relationships that could have appeared to influence the work reported in this paper.

## Figures and Tables

**figs1:**